# *catena*-Poly[[bis­(3-methyl-4-nitro­pyridine *N*-oxide-κ*O*)cadmium(II)]-di-μ-dicyanamido-κ^4^ *N* ^1^:*N* ^5^]

**DOI:** 10.1107/S1600536808044000

**Published:** 2009-01-08

**Authors:** Rong-Min Wei

**Affiliations:** a Department of Chemistry, Dezhou University, Dezhou Shandong 253023, People’s Republic of China

## Abstract

In the title compound, [Cd(C_2_N_3_)_2_(C_6_H_6_N_2_O_3_)_2_]_*n*_, the Cd^II^ ion (site symmetry 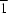) adopts a distorted trans-CdO_2_N_4_ octa­hedral environment, being coordinated by two O-bonded 3-methyl-4-nitro­pyridine *N*-oxide ligands and four dicyanamide (dca) anions. The bridging dca anions lead to a polymeric chain propagating in [100].

## Related literature

For related structures, see: Ghoshal *et al.* (2004 ▶); Wu *et al.* (2004 ▶); Schlueter *et al.* (2005 ▶).
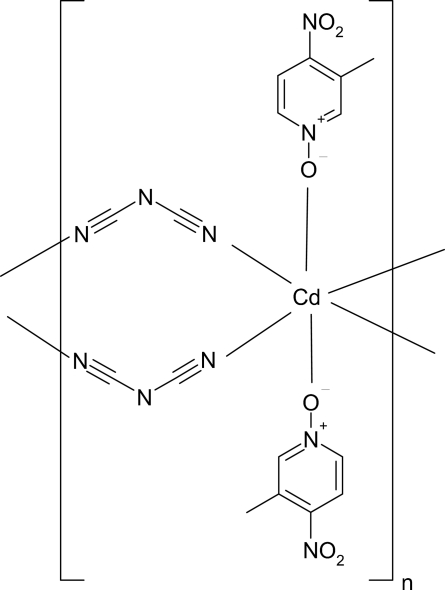


## Experimental

### 

#### Crystal data

[Cd(C_2_N_3_)_2_(C_6_H_6_N_2_O_3_)_2_]
*M* *_r_* = 552.76
Triclinic, 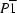

*a* = 7.5472 (8) Å
*b* = 7.5606 (8) Å
*c* = 9.8352 (10) Å
α = 83.680 (1)°
β = 68.528 (1)°
γ = 79.639 (1)°
*V* = 513.14 (9) Å^3^
*Z* = 1
Mo *K*α radiation
μ = 1.12 mm^−1^
*T* = 293 (2) K
0.32 × 0.22 × 0.18 mm

#### Data collection

Bruker SMART CCD area-detector diffractometer
Absorption correction: multi-scan (*SADABS*; Sheldrick, 1996 ▶) *T*_min_ = 0.692, *T*_max_ = 0.817
2770 measured reflections
1780 independent reflections
1764 reflections with *I* > 2σ(*I*)
*R*_int_ = 0.015

#### Refinement

*R*[*F* ^2^ > 2σ(*F* ^2^)] = 0.020
*wR*(*F* ^2^) = 0.053
*S* = 1.00
1780 reflections
152 parameters
H-atom parameters constrained
Δρ_max_ = 0.34 e Å^−3^
Δρ_min_ = −0.38 e Å^−3^

### 

Data collection: *SMART* (Bruker, 1998 ▶); cell refinement: *SAINT* (Bruker, 1998 ▶); data reduction: *SAINT*; program(s) used to solve structure: *SHELXS97* (Sheldrick, 2008 ▶); program(s) used to refine structure: *SHELXL97* (Sheldrick, 2008 ▶); molecular graphics: *SHELXTL* (Sheldrick, 2008 ▶); software used to prepare material for publication: *SHELXTL*.

## Supplementary Material

Crystal structure: contains datablocks global, I. DOI: 10.1107/S1600536808044000/hb2881sup1.cif

Structure factors: contains datablocks I. DOI: 10.1107/S1600536808044000/hb2881Isup2.hkl

Additional supplementary materials:  crystallographic information; 3D view; checkCIF report

## Figures and Tables

**Table 1:** Selected geometric parameters (Å, °)

Cd1—N3^i^	2.288 (2)
Cd1—N1	2.309 (2)
Cd1—O3	2.3110 (19)

Symmetry code: (i) 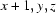.

**Fig. 1.**
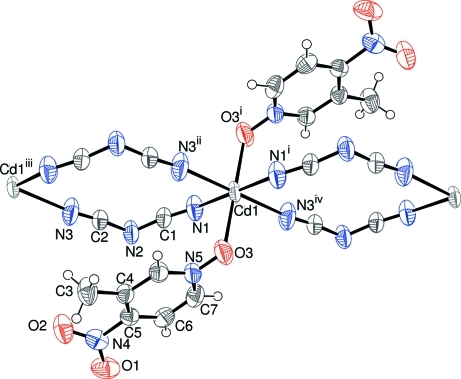
Fagment of the infinite chain structure in (I) showing 50% displacement ellipsoids for the non-hydrogen atoms. Symmetry codes: (i) 1–x, 1–y, 1–z; (ii) –x, 1–y, 1–z; (iii) x–1, y, z; (iv) 1+x, y, z.

## supplementary crystallographic information

### Comment

The pseudohalide ligand dicyanamide (dca) has been used widely due to its polydentate character and bridging ability, yielding a variety of structures and interesting magnetic properties (Ghoshal *et al.*, 2004; Wu *et al.*, 2004; Schlueter *et al.*, 2005). As a further study of such complexes, the title Cd^II^ complex, (I), is reported in this paper (Fig. 1).

Each Cd^II^ atom exhibits a slightly distorted octahedral environment with four nitrogen atoms from dicyanamide groups in the equatorial plane, and two oxygen atoms from two N-oxide (pom) ligands at the axial positions (Table 1).  Each Cd^II^ atom is coordinated to each other by the double bridging –NC—N—CN– ligands to form a one-dimensional chain structure, the Cd···Cd separation being equal to the value of the a-axis.

### Experimental

5 ml of a methanol solution of cadmium(II) chloride tetrahydrate (0.5 mmol, 128 mg) and 5 ml of a methanol sulution of dicyanamide (1 mmol, 170 mg) were aded to 10 ml of a methanol solution of POM (1 mmol, 154 mg). The mixture was stirred for 2 h and filtered. The filtrate was slowly evaporated at room temperture and red blocks of (I) were obtained after three weeks.

### Refinement

The hydrogen atoms were included in calculated positions (C—H = 0.93–0.96Å) and refined as riding with *U*_iso_(H) = 1.2*U*_eq_(C) or 1.5U_eq_(methyl C).

### Crystal data

**Table:**

[Cd(C_2_N_3_)_2_(C_6_H_6_N_2_O_3_)_2_]	*Z* = 1
*M**_r_* = 552.76	*F*(000) = 274
Triclinic, *P*1	*D*_x_ = 1.789 Mg m^−^^3^*D*_m_ = 1.789 Mg m^−^^3^*D*_m_ measured by not measured
Hall symbol: -P 1	Mo *K*α radiation, λ = 0.71073 Å
*a* = 7.5472 (8) Å	Cell parameters from 2499 reflections
*b* = 7.5606 (8) Å	θ = 2.7–27.9°
*c* = 9.8352 (10) Å	µ = 1.12 mm^−^^1^
α = 83.680 (1)°	*T* = 293 K
β = 68.528 (1)°	Block, red
γ = 79.639 (1)°	0.32 × 0.22 × 0.18 mm
*V* = 513.14 (9) Å^3^	

### Data collection

**Table:**

Bruker SMART CCD area-detector diffractometer	1780 independent reflections
Radiation source: fine-focus sealed tube	1764 reflections with *I* > 2σ(*I*)
graphite	*R*_int_ = 0.015
φ and ω scans	θ_max_ = 25.0°, θ_min_ = 2.2°
Absorption correction: multi-scan (*SADABS*; Sheldrick, 1996)	*h* = −8→5
*T*_min_ = 0.692, *T*_max_ = 0.817	*k* = −8→8
2770 measured reflections	*l* = −11→11

### Refinement

**Table:**

Refinement on *F*^2^	Primary atom site location: structure-invariant direct methods
Least-squares matrix: full	Secondary atom site location: difference Fourier map
*R*[*F*^2^ > 2σ(*F*^2^)] = 0.020	Hydrogen site location: inferred from neighbouring sites
*wR*(*F*^2^) = 0.053	H-atom parameters constrained
*S* = 1.00	*w* = 1/[σ^2^(*F*_o_^2^) + (0.0283*P*)^2^ + 0.2723*P*] where *P* = (*F*_o_^2^ + 2*F*_c_^2^)/3
1780 reflections	(Δ/σ)_max_ = 0.001
152 parameters	Δρ_max_ = 0.34 e Å^−^^3^
0 restraints	Δρ_min_ = −0.38 e Å^−^^3^

### Special details

**Table:**

Geometry. All e.s.d.'s (except the e.s.d. in the dihedral angle between two l.s. planes) are estimated using the full covariance matrix. The cell e.s.d.'s are taken into account individually in the estimation of e.s.d.'s in distances, angles and torsion angles; correlations between e.s.d.'s in cell parameters are only used when they are defined by crystal symmetry. An approximate (isotropic) treatment of cell e.s.d.'s is used for estimating e.s.d.'s involving l.s. planes.
Refinement. Refinement of *F*^2^ against ALL reflections. The weighted *R*-factor *wR* and goodness of fit *S* are based on *F*^2^, conventional *R*-factors *R* are based on *F*, with *F* set to zero for negative *F*^2^. The threshold expression of *F*^2^ > σ(*F*^2^) is used only for calculating *R*-factors(gt) *etc*. and is not relevant to the choice of reflections for refinement. *R*-factors based on *F*^2^ are statistically about twice as large as those based on *F*, and *R*- factors based on ALL data will be even larger.

### Fractional atomic coordinates and isotropic or equivalent isotropic displacement parameters (Å^2^)

**Table:**

	*x*	*y*	*z*	*U*_iso_*/*U*_eq_	
Cd1	0.5000	0.5000	0.5000	0.04339 (10)	
O1	−0.1329 (4)	−0.2147 (3)	0.8956 (2)	0.0773 (6)	
O2	−0.3397 (3)	−0.0003 (3)	0.8532 (3)	0.0777 (6)	
O3	0.3605 (3)	0.3892 (3)	0.7372 (2)	0.0664 (5)	
N1	0.4059 (3)	0.2818 (3)	0.4054 (3)	0.0602 (5)	
N2	0.1066 (3)	0.1677 (3)	0.4552 (3)	0.0601 (6)	
N3	−0.2102 (3)	0.3289 (3)	0.4816 (3)	0.0697 (7)	
N4	−0.1834 (3)	−0.0583 (3)	0.8629 (2)	0.0528 (5)	
N5	0.2298 (3)	0.2829 (3)	0.7670 (2)	0.0474 (4)	
C1	0.2592 (3)	0.2383 (3)	0.4278 (2)	0.0417 (5)	
C2	−0.0590 (3)	0.2619 (3)	0.4680 (2)	0.0424 (5)	
C3	−0.3067 (4)	0.3407 (4)	0.9163 (4)	0.0644 (7)	
H3A	−0.3083	0.4579	0.9460	0.097*	
H3B	−0.3774	0.2705	0.9994	0.097*	
H3C	−0.3652	0.3524	0.8433	0.097*	
C4	−0.1028 (3)	0.2488 (3)	0.8542 (2)	0.0416 (5)	
C5	−0.0425 (3)	0.0661 (3)	0.8317 (2)	0.0404 (5)	
C6	0.1501 (4)	−0.0059 (3)	0.7798 (2)	0.0481 (5)	
H6	0.1871	−0.1290	0.7677	0.058*	
C7	0.2853 (4)	0.1047 (4)	0.7467 (3)	0.0529 (6)	
H7	0.4155	0.0579	0.7101	0.063*	
C8	0.0426 (3)	0.3523 (3)	0.8193 (3)	0.0466 (5)	
H8	0.0092	0.4754	0.8326	0.056*	

### Atomic displacement parameters (Å^2^)

**Table:**

	*U*^11^	*U*^22^	*U*^33^	*U*^12^	*U*^13^	*U*^23^
Cd1	0.02232 (12)	0.04541 (15)	0.06540 (16)	−0.00754 (9)	−0.01648 (10)	−0.00752 (10)
O1	0.1114 (18)	0.0440 (10)	0.0825 (14)	−0.0268 (11)	−0.0370 (13)	0.0064 (9)
O2	0.0531 (12)	0.0775 (14)	0.1087 (17)	−0.0215 (10)	−0.0273 (11)	−0.0157 (12)
O3	0.0540 (11)	0.0882 (14)	0.0684 (11)	−0.0361 (10)	−0.0250 (9)	0.0028 (10)
N1	0.0430 (12)	0.0582 (12)	0.0873 (16)	−0.0097 (10)	−0.0270 (11)	−0.0188 (11)
N2	0.0338 (11)	0.0471 (11)	0.1011 (17)	−0.0055 (9)	−0.0260 (11)	−0.0039 (11)
N3	0.0386 (13)	0.0663 (14)	0.109 (2)	0.0053 (11)	−0.0341 (12)	−0.0165 (13)
N4	0.0665 (15)	0.0482 (12)	0.0449 (10)	−0.0183 (10)	−0.0151 (9)	−0.0078 (8)
N5	0.0407 (11)	0.0577 (12)	0.0483 (10)	−0.0143 (9)	−0.0189 (8)	0.0006 (8)
C1	0.0333 (12)	0.0389 (11)	0.0559 (12)	−0.0019 (9)	−0.0185 (9)	−0.0114 (9)
C2	0.0361 (13)	0.0462 (11)	0.0487 (12)	−0.0063 (9)	−0.0181 (9)	−0.0071 (9)
C3	0.0403 (14)	0.0534 (14)	0.094 (2)	0.0019 (11)	−0.0176 (13)	−0.0192 (13)
C4	0.0370 (11)	0.0405 (11)	0.0470 (11)	−0.0040 (9)	−0.0147 (9)	−0.0043 (9)
C5	0.0450 (12)	0.0398 (11)	0.0375 (10)	−0.0078 (9)	−0.0152 (9)	−0.0018 (8)
C6	0.0527 (14)	0.0429 (12)	0.0475 (12)	0.0049 (10)	−0.0206 (10)	−0.0075 (9)
C7	0.0383 (12)	0.0652 (15)	0.0529 (13)	0.0042 (11)	−0.0174 (10)	−0.0087 (11)
C8	0.0446 (13)	0.0395 (11)	0.0573 (13)	−0.0061 (9)	−0.0201 (10)	−0.0028 (9)

### Geometric parameters (Å, °)

**Table:**

Cd1—N3^i^	2.288 (2)	N4—C5	1.472 (3)
Cd1—N3^ii^	2.288 (2)	N5—C8	1.341 (3)
Cd1—N1	2.309 (2)	N5—C7	1.351 (3)
Cd1—N1^iii^	2.309 (2)	C3—C4	1.498 (3)
Cd1—O3^iii^	2.3110 (19)	C3—H3A	0.9600
Cd1—O3	2.3110 (19)	C3—H3B	0.9600
O1—N4	1.221 (3)	C3—H3C	0.9600
O2—N4	1.216 (3)	C4—C8	1.381 (3)
O3—N5	1.314 (3)	C4—C5	1.390 (3)
N1—C1	1.149 (3)	C5—C6	1.379 (3)
N2—C1	1.283 (3)	C6—C7	1.360 (4)
N2—C2	1.292 (3)	C6—H6	0.9300
N3—C2	1.128 (3)	C7—H7	0.9300
N3—Cd1^iv^	2.288 (2)	C8—H8	0.9300
			
N3^i^—Cd1—N3^ii^	180.0	C8—N5—C7	120.7 (2)
N3^i^—Cd1—N1	87.09 (8)	N1—C1—N2	172.1 (2)
N3^ii^—Cd1—N1	92.91 (8)	N3—C2—N2	173.4 (3)
N3^i^—Cd1—N1^iii^	92.91 (8)	C4—C3—H3A	109.5
N3^ii^—Cd1—N1^iii^	87.09 (8)	C4—C3—H3B	109.5
N1—Cd1—N1^iii^	180.0	H3A—C3—H3B	109.5
N3^i^—Cd1—O3^iii^	91.11 (9)	C4—C3—H3C	109.5
N3^ii^—Cd1—O3^iii^	88.89 (9)	H3A—C3—H3C	109.5
N1—Cd1—O3^iii^	87.78 (8)	H3B—C3—H3C	109.5
N1^iii^—Cd1—O3^iii^	92.22 (8)	C8—C4—C5	115.5 (2)
N3^i^—Cd1—O3	88.89 (9)	C8—C4—C3	117.9 (2)
N3^ii^—Cd1—O3	91.11 (9)	C5—C4—C3	126.5 (2)
N1—Cd1—O3	92.22 (8)	C6—C5—C4	121.8 (2)
N1^iii^—Cd1—O3	87.78 (8)	C6—C5—N4	117.4 (2)
O3^iii^—Cd1—O3	180.0	C4—C5—N4	120.8 (2)
N5—O3—Cd1	119.76 (14)	C7—C6—C5	119.4 (2)
C1—N1—Cd1	132.98 (19)	C7—C6—H6	120.3
C1—N2—C2	123.0 (2)	C5—C6—H6	120.3
C2—N3—Cd1^iv^	172.3 (2)	N5—C7—C6	119.8 (2)
O2—N4—O1	124.5 (2)	N5—C7—H7	120.1
O2—N4—C5	118.6 (2)	C6—C7—H7	120.1
O1—N4—C5	116.8 (2)	N5—C8—C4	122.8 (2)
O3—N5—C8	119.5 (2)	N5—C8—H8	118.6
O3—N5—C7	119.7 (2)	C4—C8—H8	118.6
			
N3^i^—Cd1—O3—N5	68.54 (19)	C3—C4—C5—C6	−177.0 (2)
N3^ii^—Cd1—O3—N5	−111.46 (19)	C8—C4—C5—N4	−179.23 (19)
N1—Cd1—O3—N5	−18.51 (19)	C3—C4—C5—N4	2.9 (4)
N1^iii^—Cd1—O3—N5	161.49 (19)	O2—N4—C5—C6	−151.9 (2)
O3^iii^—Cd1—O3—N5	−103 (100)	O1—N4—C5—C6	27.3 (3)
N3^i^—Cd1—N1—C1	−34.0 (3)	O2—N4—C5—C4	28.2 (3)
N3^ii^—Cd1—N1—C1	146.0 (3)	O1—N4—C5—C4	−152.6 (2)
N1^iii^—Cd1—N1—C1	139 (100)	C4—C5—C6—C7	−1.5 (3)
O3^iii^—Cd1—N1—C1	−125.2 (3)	N4—C5—C6—C7	178.7 (2)
O3—Cd1—N1—C1	54.8 (3)	O3—N5—C7—C6	178.9 (2)
Cd1—O3—N5—C8	−97.0 (2)	C8—N5—C7—C6	−0.2 (3)
Cd1—O3—N5—C7	84.0 (2)	C5—C6—C7—N5	1.1 (3)
Cd1—N1—C1—N2	−128.5 (19)	O3—N5—C8—C4	−179.4 (2)
C2—N2—C1—N1	174.7 (18)	C7—N5—C8—C4	−0.4 (3)
Cd1^iv^—N3—C2—N2	−173.9 (15)	C5—C4—C8—N5	0.1 (3)
C1—N2—C2—N3	−180 (100)	C3—C4—C8—N5	178.1 (2)
C8—C4—C5—C6	0.9 (3)		

Symmetry codes: (i) −*x*, −*y*+1, −*z*+1; (ii) *x*+1, *y*, *z*; (iii) −*x*+1, −*y*+1, −*z*+1; (iv) *x*−1, *y*, *z*.
